# Clinician based decision tool to guide recommended interval between colonoscopies: development and evaluation pilot study

**DOI:** 10.1186/s12911-022-01872-z

**Published:** 2022-05-17

**Authors:** Leigh Anne Shafer, Gayle Restall, Alexandria Simms, Eugene Lee, Jason Park, Harminder Singh

**Affiliations:** 1 Section of Gastroenterology, Department of Internal Medicine, Rady Faculty of Health Sciences, University of Manitoba, 805-715 McDermot Avenue, Winnipeg, MB R3E3P4 Canada; 2 Department of Community Health Sciences, Rady Faculty of Health Sciences, University of Manitoba, Winnipeg, MB Canada; 3 CancerCare Manitoba Research Institute, Winnipeg, MB Canada; 4 Department of Occupational Therapy, Rady Faculty of Health Sciences, University of Manitoba, Winnipeg, Canada

**Keywords:** Colorectal cancer, Guidelines, Colon polyps

## Abstract

**Background:**

Optimal intervals between repeat colonoscopies could improve patient outcomes and reduce costs. We evaluated: (a) concordance between clinician and guideline recommended colonoscopy screening intervals in Winnipeg, Manitoba, (b) clinician opinions about the utility of an electronic decision-making tool to aid in recommending screening intervals, and (c) the initial use of a decision-making smartphone/web-based application.

**Methods:**

Clinician endoscopists and primary care providers participated in four focus groups (N = 22). We asked participating clinicians to evaluate up to 12 hypothetical scenarios and compared their recommended screening interval to those of North American guidelines. Fisher’s exact tests were used to assess differences in agreement with guidelines. We developed a decision-making tool and evaluated it via a pilot study with 6 endoscopists.

**Result:**

53% of clinicians made recommendations that agreed with guidelines in ≤ 50% of the hypothetical scenarios. Themes from focus groups included barriers to using a decision-making tool: extra time to use it, less confidence in the results of the tool over their own judgement, and having access to the information required by the tool (e.g., family history). Most were willing to try a tool if it was quick and easy to use. Endoscopists participating in the tool pilot study recommended screening intervals discordant with guidelines 35% of the time. When their recommendation differed from that of the tool, they usually endorsed their own over the guideline.

**Conclusions:**

Endoscopists are overconfident and inconsistent with applying guidelines in their polyp surveillance interval recommendations. Use of a decision tool may improve knowledge and application of guidelines. A change in practice may require that the tool be coupled with continuing education about evidence for improved outcomes if guidelines are followed.

**Supplementary Information:**

The online version contains supplementary material available at 10.1186/s12911-022-01872-z.

## Introduction

Colorectal cancer (CRC) remains the second most common cause of cancer related deaths in North America [1, 2]. Most CRCs are known to develop from colorectal polyps Removal of colorectal polyps has been shown to decrease the development of CRC and subsequent related deaths [3–7].

Colonoscopy has become one of the most common medical procedures due to increase in CRC screening and surveillance of patients with colorectal polyps. Due to rising numbers of colonoscopies, wait times for the procedure have been increasing in Canada [8, 9]. Furthermore, wait times for colonoscopies have recently risen even more sharply due to cancellations of non-emergent procedures during  the COVID pandemic.

The estimated risk for development of CRC varies by family history of colorectal cancer, and size, number, and histology of colorectal polyps. Common histological features of colorectal polyps include villous or tubular adenomas, high grade or low-grade dysplasia, sessile serrated polyp, sessile serrated polyp with dysplasia, hyperplastic polyp, and traditional serrated adenomas [10–14]. North American guidelines [15–17] take many factors into account in their recommended colonoscopy surveillance interval [10–12, 18]. Factors include age, family history of CRC and advanced adenomas, and presence, number, size, and type of polyps present in both current and previous colonoscopy. The breadth of factors to consider sometimes makes it difficult for an individual clinician to recall and apply [18–21]. In addition, recommendations originating from outside of North America [22] differ in subtle ways from those developed locally and add to confusion for the clinician. Finally, the most recent North American guidelines have added categories, making them potentially more difficult to implement in clinical practice [23]. For example, in the new guidelines, specific recommendations after removal of 3–4 adenomas < 10 mm in size and detailed recommendations for follow-up after removal of different categories of serrated polyps have been provided.

Several studies have demonstrated a high frequency of repeat colonoscopies at shorter and longer intervals than those recommended in guidelines [21, 24–29]. If individuals are followed at longer than recommended intervals, they are exposed to an increased risk of developing endoscopically unresectable polyps or, rarely, CRC in the interim. If individuals are followed at shorter than recommended intervals, they are exposed to the risks and inconvenience of an unnecessary colonoscopy. Shorter intervals also increase wait times for others awaiting colonoscopy and increase costs to the health care system [25, 27]. The effects of unnecessary colonoscopies on patients and the health care system have been greater during the COVID-19 pandemic, with delays occurring due to postponing out-patient procedures, including colonoscopies.

Clinical decision-making tools have been applied to guide procedures for a wide variety of health needs and have been shown to improve adherence to health care guidelines [30–33]. Although tools exist to support both patients [34] and clinicians [35] in making decisions about CRC screening intervals, we aimed to develop and evaluate a tool that would be very simple and quick to use (1 to 2 min) and that may be more widely available and implemented by clinicians.

## Methods

We used a multi-phased approach to develop and pilot test a decision support tool to guide clinicians in recommending patient-specific surveillance intervals. During the first phase we used North American CRC surveillance guidelines [15, 16, 36] and consultation with endoscopy experts to develop the decision tool. During the second phase we evaluated endoscopist and primary care provider (PCP) adherence with recommended colonoscopy screening intervals through practice scenarios. In addition, we assessed current clinician approaches to determine follow-up recommendations and obtained their feedback about the newly developed decision support tool through focus groups. Finally, we trialled the decision tool in a pilot study with endoscopists and obtained feedback on the utility of the tool in practice. Qualitative and quantitative data collection for this study was conducted in Winnipeg, Manitoba, Canada.

### Tool development

**Fig. 1:**
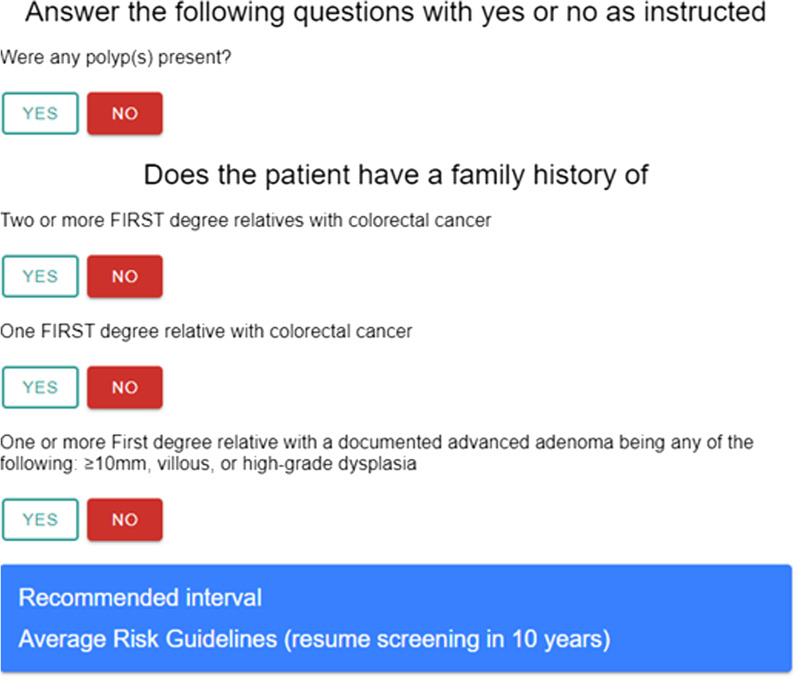
Sample page of surveillance tool—index colonoscopy

We developed the screening interval decision tool incorporating recommendations from North American guidelines. The main guidelines used were from the Canadian Association of Gastroenterology (CAG) [16, 36]. The United States multi-society task force (USMSTF) on CRC guidelines were used to fill in areas that were not addressed by the CAG guidelines [15, 37]. For example, follow-up after CRC resection was not addressed by CAG so USMSTF guidelines were used for this scenario. Upon reviewing the guidelines, a tool was created with four to thirteen questions using conditional logic. For example, if the current colonoscopy was the index colonoscopy for the patient, then the index colonoscopy page is displayed on the tool (Fig. 1). We have provided the full tool in Additional File 1: Appendix I. The number and type of questions required for the tool to provide a recommended surveillance interval depended on whether this was a first colonoscopy, a surveillance colonoscopy, or a colonoscopy after diagnosis of CRC. In pre-study testing, use of the tool took less than one minute per patient. The recommended surveillance interval is based on personal history of CRC, polyp characteristics on index and prior colonoscopy and family history of CRC and adenoma. Recommendations for individuals with hereditary CRC syndromes or IBD were not included in this tool.

### Focus groups

Convenient and purposeful sampling strategies were used to recruit participants from different specialties and levels of training, including residents: gastroenterologists (GI), surgeons who perform endoscopy, and PCPs. There were four focus groups. One surgery group was recruited with the help of one of our surgeon authors (JP) and consisted of 5 surgeons and 4 surgery residents. Two GI groups were made up of 7 GIs and 2 gastroenterology residents and had 4 or 5 participants per focus group. The GI focus groups were performed at their regularly scheduled noon hour teaching rounds and the surgery focus group after one of their service rounds. The PCP group was recruited via a Cancer Care Manitoba teaching event for PCPs (and hence with higher interest in colorectal neoplasia management) and comprised 4 practitioners. Focus groups lasted one to two hours.

At the beginning of each focus group, participants completed a demographic and clinical background questionnaire and were asked to state their recommended colonoscopy follow-up time for several scenarios (Additional File 1: Appendix II). The surgeons and surgeon residents were given 7 scenarios to assess (questions 1a-1c, and 2–5 of Additional File 1: Appendix II), while the GIs, GI residents, and PCPs were given an additional 5 scenarios (questions 6 and 7a-7d of Additional File 1: Appendix III) for 12 scenarios in total.

Following the background questionnaire and scenarios, participants engaged in a facilitated discussion about approaches to determining colonoscopy follow-up recommendations and feedback about the decision tool. The discussions were audio-recorded, transcribed verbatim, and followed a semi-structured interview format (Additional File 1: Appendix III). Topics included approaches to determining colonoscopy follow-up recommendations and methods of communicating follow-up recommendations to PCPs and patients. A prototype of the decision tool was demonstrated (Additional File 1: Appendix I) and participants were asked for their feedback about the tool.

Focus group transcripts were analysed (by AS and GR) using deductive and inductive qualitative content analysis with NVivo 11 qualitative data analysis software [38]. Each focus group was first analysed individually through line-by-line coding. The semi-structured interview questions were used as an initial framework and additional codes were added throughout the analytic process. Data were compared across the four focus groups. Participant opinions were coded, condensed into similar coded groups and then summarized for content representing overall opinions and concepts. Unique perspectives were also coded. Results were reviewed with all authors who facilitated the focus groups throughout the analysis. Revisions to the tool were made based on the focus groups, principally a web based application was developed in addition to the online version.

### Quantitative pilot study

After completion of the focus groups, 10 endoscopists were approached through purposeful convenience sampling. Six agreed (five trained in Canada and one trained in the United States, all working in Winnipeg) to participate in the pilot study to evaluate the decision tool. Two of these six had also participated in one of the focus groups. These six endoscopists agreed to complete 10 of their scheduled patient colonoscopies as part of the pilot study and most completed all 10. After making their recommendation for surveillance interval for follow-up after each colonoscopy, endoscopists used the decision tool, and then completed a survey (Additional File 1: Appendix IV). The survey asked for their recommended surveillance interval for follow-up colonoscopy before they used the tool, whether their recommendation matched the decision support tool, and whether they would have changed their recommendation if they had used the tool prior to making their recommendation. In addition to the survey responses which indicated agreement or not with the tool, chart reviews were conducted to assess the accuracy of data entry into the tool.

Five of the six endoscopists who completed the tool evaluation pilot study were surveyed for their initial impressions about the utility of the tool (Additional File 1: Appendix IV, part 2). One of the six endoscopists who participated in the pilot study left after completing 7 colonoscopies and did not complete the initial impressions survey. We summarized the pilot information descriptively.

### Assessment of study objectives

Our study had three objectives:

- The concordance between clinicians and guidelines was assessed through the scenario responses This was done in theory through the scenario responses collected during focus groups. It was done in practice through the recommended intervals suggested during the pilot study.
- Assessment of clinician opinions about the utility of an electronic decision-making tool. This was done qualitatively during the focus groups and quantitatively during the pilot study. The study participants in the pilot study completed a brief follow-up survey after using the tool with 10 patients in their practices.
- Assessment of the initial use of a decision-making smartphone/web-based application. Ease of use, convenience of the tool, and initial impressions about whether the endoscopists would use the tool in practice were assessed during the pilot study.

### Ethics

This study was approved by the Health Research Ethics Board at the University of Manitoba.

## Results

In total, 22 clinicians participated in the qualitative focus group discussions, and 19 completed the pre-survey in which they stated their recommended follow-up interval for each scenario without using the decision tool. Of the 19 who completed the scenario survey, 9 (the surgeons) were given a survey with 7 scenarios, and the remaining 10 conducted the same exercise with 5 additional scenarios for a total of 12 scenarios (Table 1). Six endoscopists participated in the pilot study evaluation of the tool by using it with their respective colonoscopy patients. Most (> 70%) received their training in Canada, with the remainder receiving their training in the United States. Among focus group participants, 27% were women; no women participated in the pilot study tool evaluation. Years in practice ranged from < 1 to 9 among the residents and from < 1 to 40 + among those with completed training.

**Table 1:** Background characteristics of study participants

	Focus groups (FG)			Tool evaluation pilot study (N = 6)^b^
	Qualitative FG participation (N = 22)	Completed 7 scenario survey (N = 19)	Completed 12 scenario survey^a^ (N = 10)	
*Clinician type*				
General Surgeon	5 (23%)	5 (26%)	0	3 (50%)
Gastroenterologist	7 (32%)	4 (21%)	4 (40%)	3 (50%)
Primary Care Physician	4 (18%)	4 (21%)	4 (40%)	0
Resident (Surgeon or GI)	6 (27%)	6 (32%)	2 (20%)	0
*Training*				
Canada	16 (73%)	16 (84%)	9 (90%)	5 (83%)
U.S.A	3 (14%)	1 (5%)	1 (10%)	1 (17%)
Canada and U.S.A	3 (14%)	2 (11%)	0	0
*Gender*				
Male	16 (73%)	13 (68%)	5 (50%)	6 (100%)
Female	6 (27%)	6 (32%)	5 (50%)	0

^a^The 12 scenario survey comprised the same scenarios as the 7 scenario survey, plus an additional 5 scenarios. The General Surgeons and Surgeon Residents were given the 7-scenario version of the survey in their focus group

^b^Of the endoscopists in the pilot study, 2 were also in the focus groups while 4 did not overlap with those in the focus groups

### Qualitative focus groups

Focus group analysis identified similar and contrasting opinions among and between GIs and surgeons, and PCPs in relation to use of guidelines and impressions of the decision tool.

#### Use of guidelines

Participants in all groups reported using a variety of guidelines (e.g., American College of Gastroenterology, USMTF, CAG). However, individuals reported that they tended to use a particular guideline or two in their practice.

How often endoscopists reported checking the guidelines varied. Some reported having memorized the guidelines and only referred to them rarely or never. Others acknowledged checking more often to confirm recommended follow-up intervals. Some endoscopists reported using guidelines as a reference but adjust recommended surveillance intervals using their own clinical judgement, especially in complex clinical situations. Some reported using the guidelines primarily as a teaching tool with students.

Guidelines were not commonly used among the PCP group except when working with students, or if working in a specialized role. A participant in the PCP focus group reported that a current trend in primary care was to not trust guidelines that did not involve patient input during development and that guidelines were often perceived to be based on limited evidence and author opinions. PCPs were more likely to rely on the recommendations provided by the endoscopist.

#### Impressions of the decision tool: features

Participants in all focus groups noted the importance of the decision tool being quick and easy to use and access. Concern was noted in all groups that the tool must keep up with current evidence and should communicate updates clearly to allow users to trust the recommendations. Some clinicians felt that guidelines themselves are often dated and do not incorporate current evidence by the time they are published. Participants also identified the challenge of working with information in multiple unconnected systems and noted a preference for the decision tool to be integrated into an existing electronic medical record.

Endoscopists’ preferences were mixed for a web-based or mobile app format for the decision tool. Some endoscopists were optimistic about the tool being useful but were interested in testing the decision tool in practice before deciding. Others expressed the challenge of practice autonomy and that no matter how effective the tool was, that the endoscopists who should use it most, probably would not. Some endoscopists felt the tool would not work in all scenarios and expressed the need to clearly identify such scenarios in the tool.

PCPs suggested the decision tool could support patient-informed shared decision making about recommended surveillance intervals for colonoscopy by adding brief information that informs PCPs and patients about specific risk factors and the evidence behind recommendations. The PCP group also discussed the potential of the decision tool to improve communication of recommended surveillance intervals to patients. The tool may be particularly helpful to PCPs in rural and remote areas. The PCP group noted that polyp follow-up is often a small part of primary care practice which may limit uptake of the decision tool among PCPs.

#### Impressions of the decision tool: process challenges

A common challenge identified by focus group participants related to having easy access to all information that was needed to input into the decision tool (e.g., polyp size and type, accurate patient and family history). All groups identified shared challenges about applying decision tool recommendations in practice. Participants noted difficulties with maintaining connection with patients over follow-up times of 5–10 years. Clinicians also identified concerns about follow-up being dependent on individual clinicians versus managed centrally to prevent patients missing follow-up due to changing care providers.

When discussing who should use the tool, some endoscopists welcomed the idea of PCPs using the tool to determine or confirm guideline recommended surveillance intervals for referring patients for colonoscopy. Others perceived potential conflicts if the PCPs questioned an endoscopist’s recommendation for a patient if it did not match the guidelines.

PCPs suggested that endoscopists could use the decision tool to support and communicate their recommended interval to PCPs and patients. Some PCPs reported they may use the decision tool if they felt there was a discrepancy between the recommendation made by an endoscopist and the guidelines. Some thought the tool may give them an opportunity to communicate with the endoscopist to understand additional factors that led to a non-guideline recommendation.

Discussion about who should use the decision tool led to all groups identifying challenges regarding role confusion and who is responsible for communicating results to the patient and tracking and scheduling follow-up colonoscopy. Endoscopists differed on assigning responsibility for tracking patient follow-up to the endoscopist, PCP, patient, or combination of all three. PCPs often assumed results and follow-up recommendations had been communicated by endoscopists to patients directly unless specifically communicated otherwise. However, PCPs reported often being unclear as to who was expected to track and schedule the follow-up colonoscopy.

### Quantitative evaluation of knowledge of colonoscopy follow-up interval guidelines

Of the 10 participants who provided during focus groups their recommended surveillance interval in 12 scenarios, the highest number consistent with current guidelines was 10 out of 12 (83%) by one participant, while the lowest was 3 out of 12 (25%) by one participant (Table 2). The two incorrect responses by the participant with 83% correct response were on questions 4 (the correct interval was 5 years while the participant suggested 3 years) and 7a (the correct interval was 5 years while the participant skipped this question). Refer to Additional File 1: Appendix II for scenario questions. Five participants (50%) scored 6 or fewer (50% or below) correct in the 12 scenarios (Table 2). Of the 19 participants who completed the 7 scenarios, the highest score was 6 out of 7 by one participant, while the lowest was 0/7 by one participant. Overall, 10/19 scored below 50% on these 7 scenarios (Table 2). There were no statistically significant differences between clinician type (GI, surgeons, or PCP) in the percent who scored <  = 50% correct nor between genders, either among the 12 scenarios or among the 7 scenarios. Majority (58%) of the incorrect responses were for longer follow up intervals than that recommended in the guidelines.

**Table 2:** Evaluation of clinician knowledge of colonoscopy follow-up interval guidelines

	Number (%) of clinicians who scored 0–3 correct out of 7 scenarios (< = 50% correct)	*p*-value^a^	Number (%) of clinicians who scored 0–6 correct out of 12 scenarios (< = 50% correct)	*p*-value^a^
*Clinician type*				
General Surgeon	2/5 (40%)	0.86	NA	> 0.99
Gastroenterologist	2/4 (50%)		2/4 (50%)	
Primary Care Physician	3/4 (75%)		2/4 (50%)	
Resident (Surgeon or GI)	3/6 (50%)		1/2 (50%)	
*Gender*				
Male	6/13 (46%)	0.63	3/5 (60%)	0.50
Female	3/6 (50%)		2/5 (40%)	
Total	10/19 (53%)		5/10 (50%)	

^a^Fisher’s exact test, comparing percent correct across clinician types

### Quantitative evaluation of decision tool for recommended colonoscopy surveillance interval

The decision tool was used and evaluated in 58 total colonoscopies by the 6 endoscopists who participated in the pilot study evaluation (four completed 10, one completed 11, and one completed 7). Agreement between endoscopist recommended surveillance interval and tool recommended surveillance interval was higher in the pilot study among patients in practice, than it was in the hypothetical cases evaluated during the focus groups. Agreement during the pilot study was 65%, excluding the cases where endoscopists made guideline “discordant” recommendations because of patient age or cases not covered in the tool (hereditary condition/anal cancer) (Table 3). During the pilot study, issues arose in 12 out of the 58 colonoscopies (20%) on which the tool was used (Table 3). In four instances, the endoscopist stated that there was agreement in the recommended follow-up time, but a subsequent chart review suggested that there was not agreement. In eight instances, the endoscopist may have been correct, even though their recommendation seemingly did not agree with that of the tool. Six of these eight instances involved age of the patient, in which the endoscopist recommended no further follow-up because the patient was old (the tool is not intended to be used for patients over age 75) and the other two involved hereditary colorectal syndrome/anal cancer. Most (70%) of the discordant responses among the 46 colonoscopies with no issues, were for shorter follow up intervals than that recommended in the guidelines.

**Table 3:** Pilot study evaluation of decision tool

Did endoscopist recommendation agree with tool?		
	All colonoscopies (N = 58)	Excluding colonoscopies with incorrect tool use (N = 46)
No	16 (28%)	16 (35%)
Yes	30 (52%)	30 (65%)
Incorrectly used tool (stated agreement but chart review suggests disagreement)	4 (7%)	–
Endoscopist recommended no follow-up because of age but tool recommended f/u	6 (10%)	–
Other reason that endoscopist right/tool not applicable (hereditary syndrome/anal cancer)	2 (3%)	–

Endoscopist evaluation of tool (N = 5 endoscopists)^a^							
Tool reliable		Tool easy to use		Tool information new or familiar		Would use tool in practice	
Strongly disagree	0	Strongly disagree	0	Very familiar	1	Very unlikely	1
Disagree	0	Disagree	0	Familiar	0	Unlikely	2
Neutral	0	Neutral	0	Unsure	0	Neutral	1
Agree	3	Agree	2	New	2	Likely	1
Strongly agree	2	Strongly agree	3	Very new	2	Very Likely	0

^a^One of the six endoscopists in the pilot study did not complete the final tool evaluation

All five participants who completed the survey about the utility of the tool agreed or strongly agreed that the tool was both reliable and easy to use, and four of the five stated that the information from the tool was new or very new (Table 3). Although endoscopists and PCPs stated in focus groups that they would find a decision tool useful, three of five who evaluated the tool in the pilot study stated that they would be unlikely or very unlikely to use the evaluated tool. This was despite incongruent recommendations being given 35% of the time without an obvious reason. In focus groups, endoscopists thought that the tool might be helpful for more complicated situations or when they were unsure of the guidelines. PCPs felt the tool would be helpful for most situations but that they may not have time to use it with every patient. Both groups felt they would be more likely to use the tool if the it was easy to use and did not require too much time. This was borne out in our pilot study.

## Discussion

The results of both the qualitative and quantitative data in our study suggest that endoscopists may be overconfident in their recommendations of polyp surveillance intervals. Although their recommended intervals were often different than that in the guidelines, they were confident in their knowledge and practice and did not believe they needed to reference guideline recommendations or the studied tool. Our finding about overconfidence in endoscopist recommendations has also been found in other settings. For example, in a recent United States based study, 38% of endoscopist recommended surveillance intervals were not guideline concordant [39]. In another study, consistency between endoscopist- and guideline-recommended follow-up varied by quality of bowel preparation. Inconsistent recommendations were observed in 15% of patients with excellent preparation, 75% of patients with fair preparation, and 32% of patients with poor preparation [40].

In the hypothetical scenarios, roughly half of clinicians recommended intervals that were discordant with the most recent guidelines in over half of the scenarios. Agreement with guidelines was better in the pilot study among actual patients, but even there, the clinician recommended interval did not match those of the guidelines approximately 40% of the time. In the focus group analysis, some indicated that guidelines were dated or that they did not replace their own clinical acumen. If clinicians believe that the guidelines do not represent best practice, this suggests that a decision tool to aid in the memory of guidelines for endoscopists will not be sufficient to change practice. It is possible that the decision tool would need to be used in conjunction with further training including discussion about the value of following the guidelines. A study may be warranted to explore the interactive effect of both training and the decision tool in improving guideline adherence. In addition, given that recommendations do change over time as more research results become available, it is imperative that the decision tool be regularly updated and clearly identify when updates have been made to support clinician confidence in the tool. The results from our data echo previous studies that have shown a wide range of recommendations by clinicians for the same scenarios [21, 24, 25].

There are several potential reasons for clinician practice variation from the guideline recommendations, including lack of knowledge about guideline recommendations [24], disagreement with guideline recommendations [24] and awareness of alternate/other guideline recommendations. Disagreement with colonoscopy follow-up guideline recommendations might be due to awareness of effect of improved colonoscopy quality on reduced CRC risk or suboptimal index colonoscopy judged by lack of completion to cecum or inadequate bowel preparation. However, guideline recommendations as well as our scenarios account for and are intended for high quality complete colonoscopy with adequate bowel preparation. In addition, clinicians might be influenced by guidelines from other jurisdictions such as Europe; anecdotally, however to best of our knowledge, clinicians in Manitoba are not influenced by European colorectal screening practices and no one referred to European guidelines in the focus group discussions. Finally, as with any guideline, individual clinicians may view the evidence base differently than the guideline authors, even though most individual clinicians would not have reviewed the evidence base, and the authors of the colonoscopy follow up guidelines are well known experts in the area.

Interestingly, the main points of contention amongst primary care providers and endoscopists were not things that a decision tool might help with. They disagreed about whose responsibility it was to ensure that patient intervals were adhered to. Previous research has identified the complex and inconsistent nature of the communication among endoscopists, PCPs and patients [41]. Results from the current study support the development of standardized processes for communication and tracking of surveillance colonoscopies.

Both endoscopists and PCPs expressed concern in the focus groups about accessing the information needed for obtaining the recommended intervals from the tool. In Manitoba, as elsewhere, there are multiple interfaces that have information, and it can be difficult to navigate them to find the correct data. A single electronic medical record (EMR) could potentially help alleviate the struggle to find information and this may be demonstrated with further research in places such as Alberta [42, 43], where a single EMR system is being implemented province-wide.

In general, the agreement between the endoscopist and the tool was much higher in the pilot study conducted with real colonoscopy patients than it was in the scenario surveys. Nonetheless, there were significant disagreements in the pilot study. In some cases, the clinician may not have recalled the guidelines correctly or may disagree with the guidelines. In some cases, the disagreements between the endoscopist and tool recommended intervals resulted from the tool being used with a population that it was not intended for. This latter finding has emphasized the need for clearer instructions on tool use. For example:

1. The tool is intended for patients in the screening age group (< 75 years). A number of endoscopist recommendations differed from that of the tool because the endoscopist recommended no further colonoscopy for their older patient. In practice, clinicians will be instructed that screening decisions for older individuals should be made case by case.
2. The decision tool used in the pilot is not intended for patients with inherited malignancies. For this reason, one endoscopist recommended an interval of one year for a patient while the tool recommended 5–10 years. The tool instructions will be modified to specify that it is not intended to be used for those with hereditary cancer conditions or IBD.
3. In one case, the endoscopist recommended a shorter interval (3 years) than the tool because the tool did not account for anal adenocarcinoma follow-up. This specific scenario needs case by case decision. This anal cancer likely arose from the rectum and hence needed follow up similar to rectal cancers.

Among the 12 instances in the pilot study in which the endoscopist’s recommendation disagreed with guidelines, only twice (17% of the time) did the endoscopist state that they would have changed their recommendation if they had used the tool first. This suggests that there needs to be greater dissemination and discussion of the guidelines. It is possible that focussing efforts to improve adherence to guidelines among non-endoscopists may have a larger impact than efforts among endoscopists.

### Study strengths and limitations

The main strength of our study was the iterative process for developing the surveillance tool. We first developed the tool. Next, during focus groups we obtained feedback and discussed enhancements to the tool with people from the profession who would use the tool. After revision of the tool following the focus groups, we finally pilot tested the tool among endoscopists in practice. We felt that this iterative process of tool development would lead to buy-in and improve uptake of tool use among the clinicians.

One limitation may be gender representation. Although the gender distribution of participants in the focus groups closely resembled the gender distribution of Canadian GIs (Jewaid [44] et al. suggest 31% of Canadian GIs are women and 27% of our focus group participants were women), women were not represented at all in our pilot study, evaluating the surveillance tool in practice. This could suggest a representation bias. However, women and men displayed similar clinical knowledge of surveillance intervals when assessing scenarios during focus groups (Table 2); so, they may also have similar experiences with the tool in practice. This is an observational pilot study, and the tool ideally needs to be further evaluated in a randomized trial.

## Conclusion

Our study suggests that endoscopists are overconfident in their polyp surveillance interval recommendations. There needs to be clear communication and role definition among PCPs and endoscopists regarding responsibility for communicating results and scheduling follow-up surveillance for patients, as there is currently misalignment in the current perceived roles. Endoscopists and PCPs agreed that a decision tool may be beneficial if it was quick and easy to use. However, whether a decision tool will be used in clinical practice and, more importantly, change relevant clinical practice will need to be determined in a future study.

## Supplementary Information

**Additional file 1.** Colonoscopy screening interval tool.

## Data Availability

The datasets used and/or analysed during the current study are available from the corresponding author on reasonable request, including obtaining approval from the ethics board.
