# Supplementary material for: Clinician based decision tool to guide recommended interval between colonoscopies: development and evaluation pilot study
Source: BMC Med Inform Decis Mak. 2022 May 17;22:136. doi: 10.1186/s12911-022-01872-z (PMC9112638; doi:10.1186/s12911-022-01872-z)
Supplement: Supplementary file 1 — Additional file 1. Colonoscopy screening interval tool. [file 12911_2022_1872_MOESM1_ESM.docx]

**Appendices - Decision Tool, Guided Focus Group Script, and Surveys**

This document is composed of four appendices:

Appendix I – Colonoscopy Screening Interval Tool (p. 1)

Appendix II - Focus Group Script and Questions (p. 7)

Appendix III – Colonoscopy follow-up time scenarios (p. 10)

Appendix IV – Survey of Surveillance Tool Utility in Practice (p. 13)

**Appendix I**

**Colonoscopy Screening Interval Tool**

Each successive question on the tool that is asked is dependent on responses to previous questions. In the simplest scenario (no identified elevated risk through polyps or family history), four questions are asked and a recommendation is given. In the scenario requiring the most information, a maximum of 13 questions are asked. Sample pages from the tool follow.

Page 1


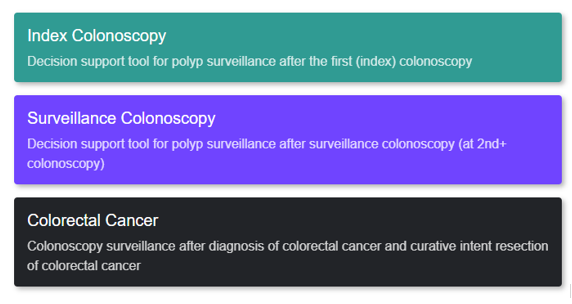


Index Colonoscopy Page

*If responses to four questions are no, then there is enough information for recommended interval:*


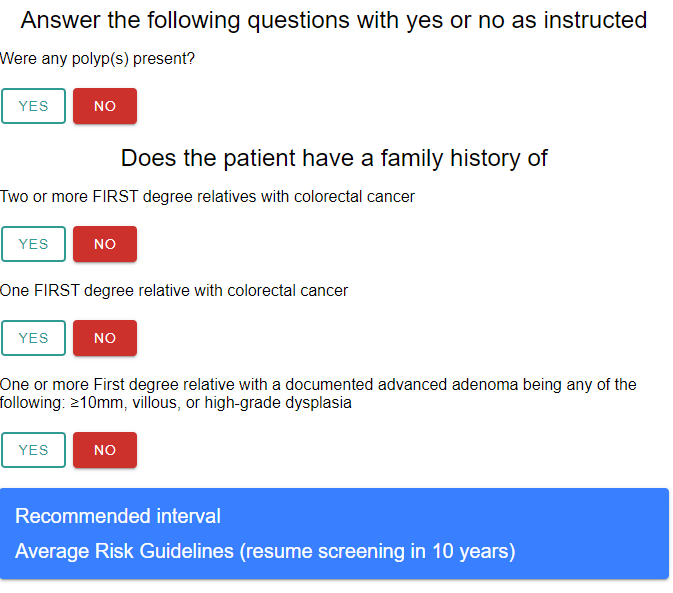


*If any response to first four questions is yes, then follow-up questions are asked:*


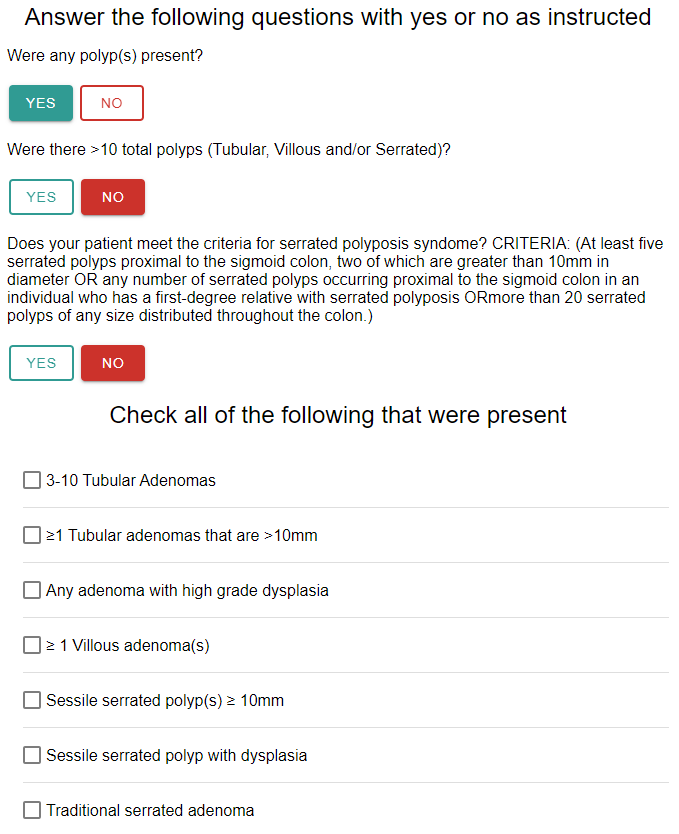


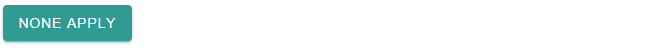


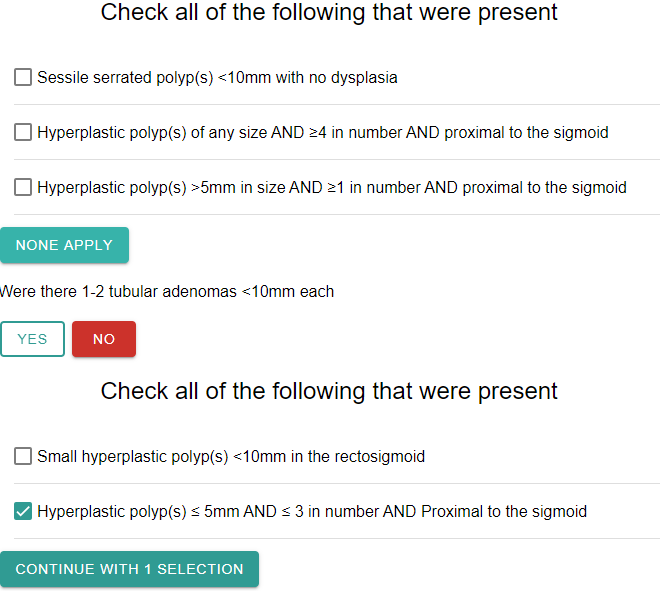


*Depending on responses to polyp questions, one or more family history questions may be asked:*


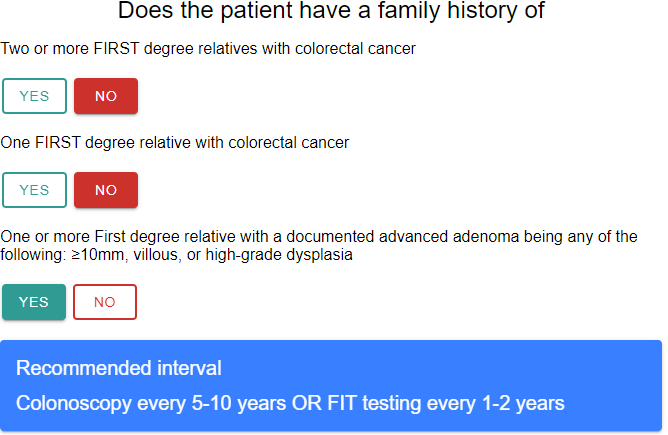


Surveillance Colonoscopy Page

*If enough information to make a recommendation is provided through current and previous colonoscopy polyp questions, then no family history is asked:*


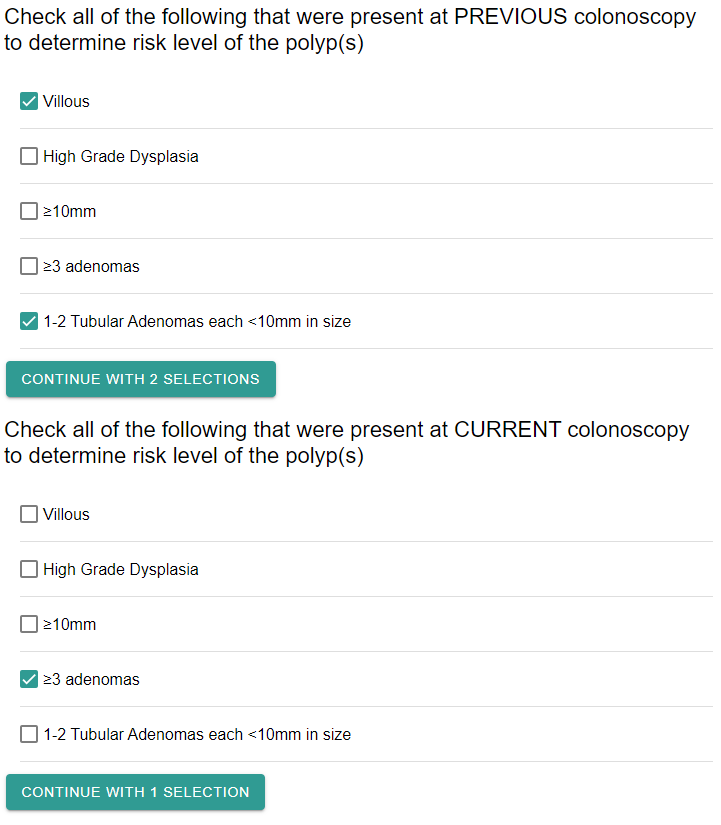


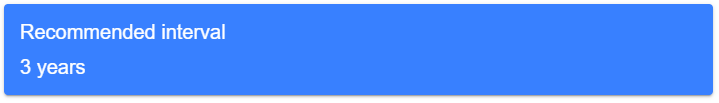


*Otherwise (if none of the above polyp situations apply), then the same family history as for the index colonoscopy is gathered, before making a recommendation.*

Colorectal Cancer Page

*
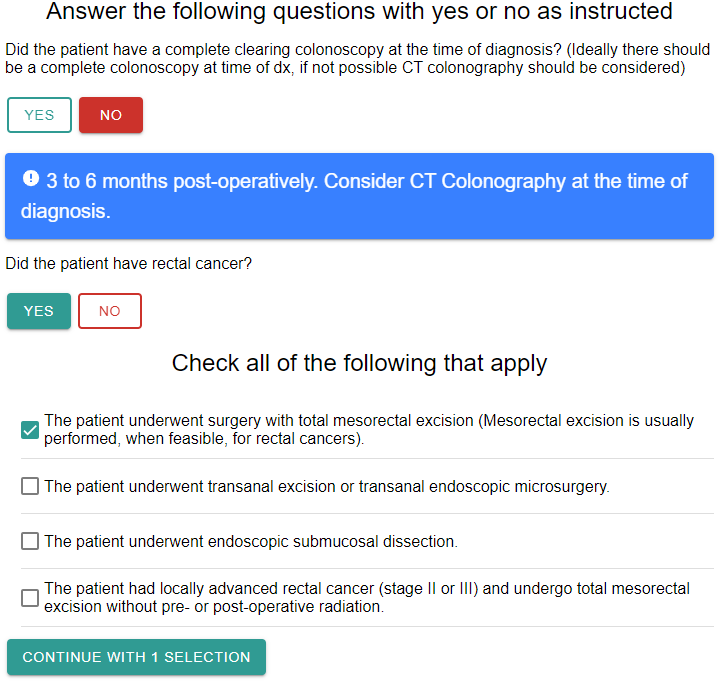
*

*
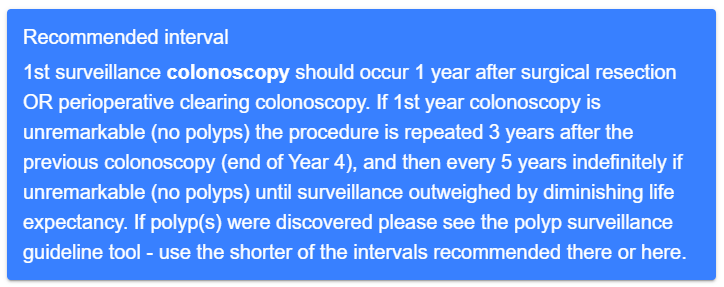
*

**Appendix II**

**Colonoscopy follow-up time scenarios**

**Scenarios – Please Circle your Answer**

1. When would you recommend follow-up colonoscopy for a 55 year old otherwise healthy women with no family history of colorectal cancer who has on her first colonoscopy:
   1. a single 5 mm tubular adenoma?
      1. 5 years
      2. 10 years
      3. 5-10 years
      4. 3 years
      5. 1 year
   2. three 5 mm tubular adenomas?
      1. 5 years
      2. 10 years
      3. 5-10 years
      4. 3 years
      5. 1 year
   3. a single 5 mm villous adenoma?
      1. 5 years
      2. 10 years
      3. 5-10 years
      4. 3 years
      5. 1 year
2. When would you recommend follow-up colonoscopy for a 60 year old otherwise healthy man with a family history of colorectal cancer in his father at age 70 and brother at age 65 who has a normal first colonoscopy
   1. 5 years
   2. 10 years
   3. 5-10 years
   4. 3 years
   5. 1 year
3. When would you recommend follow up colonoscopy for a 65 year old otherwise healthy man with no family history of colorectal cancer, who had a 1 cm villous adenoma on his first colonoscopy three years ago and now has a completely normal colonoscopy?
   1. 5 years
   2. 10 years
   3. 5-10 years
   4. 3 years
   5. 1 year
4. When would you recommend follow up colonoscopy for a 60 year old female with no family history of colorectal cancer who is found to have a sessile serrated polyp 5mm in size in the ascending colon?
   1. 5 years
   2. 10 years
   3. 5-10 years
   4. 3 years
   5. 1 year
5. What would you recommend for first follow up endoscopy for a 68-year-old male who had rectal cancer diagnosed on colonoscopy and followed shortly by removal via transanal endoscopic microsurgery?
   1. Colonoscopy 6 months post surgery
   2. Colonoscopy 1 year post surgery
   3. Colonoscopy 3 years post surgery
   4. Flexible sigmoidoscopy 3-6 months after surgery
6. When would you recommend follow up colonoscopy for a patient with no family history who on surveillance (2^nd^ c-scope) colonoscopy was found to have a 5mm tubular adenoma when the first showed a 7mm villous adenoma?
   1. 5 years
   2. 10 years
   3. 5-10 years
   4. 3 years
   5. 1 year
7. When would you recommend follow-up colonoscopy for a 55-year-old otherwise healthy man with no family history of colorectal cancer who has on his first colonoscopy:
   1. a single sessile serrated polyp?
      1. 5 years
      2. 10 years
      3. 5-10 years
      4. 3 years
      5. 1 year
   2. A sessile serrated polyp with dysplasia?
      1. 5 years
      2. 10 years
      3. 5-10 years
      4. 3 years
      5. 1 year
   3. A traditional serrated adenoma?
      1. 5 years
      2. 10 years
      3. 5-10 years
      4. 3 years
      5. 1 year
   4. A single 5 mm tubulovillous adenoma
      1. 5 years
      2. 10 years
      3. 5-10 years
      4. 3 years
      5. 1 year

**Appendix III**

**Focus Group Script and Questions**

[The following procedure will begin after obtaining informed consent from the health professionals participating in the focus group.]

Introduction:

Thanks for taking the time to join us to talk about follow up after colonoscopy procedures in Manitoba with a particular emphasis on the follow up after colon polyps.

My name is << >> and assisting me are << >> and << >>. We’re all from the University of

Manitoba.

We are working with WRHA to strengthen the system for colonoscopy and follow-up after colonoscopy in Manitoba. Colonoscopists, primary care providers and patients all have key roles in referring for colonoscopy and in follow up of the patient following colonoscopy. We want to understand the needs and perceptions of colonoscopists, primary care providers and their patients regarding these activities – with the goal of making the system as effective and efficient as possible.

Our research group has several objectives:

- To understand how current procedures function for participants in various parts of the system including patients, primary care practitioners and colonoscopists.
- To identify areas where procedures could be strengthened.
- To identify the resources that would assist physicians in managing colonoscopy procedures as effectively as possible.
- To assess the decision support tool we have developed.

Several months ago, we conducted six focus groups with colonoscopists and primary care providers. The purpose was to obtain their opinions about current practices, barriers and facilitators to following recommended practice for preparation and follow up after colonoscopy. We also obtained recommendations about, and approaches to improving the process. Our findings indicated that there were a number of communication challenges among patients, primary care providers and colonoscopists pre- and post colonoscopy. In relation to post-procedure communication, focus group participants identified a number of inconsistencies across colonoscopists in how and when results and follow-up recommendations were communicated to primary care practitioners and patients. Participants identified this as an important challenge to address. Today we would like to focus on two aspects of post colonoscopy follow-up. First, we would like to better understand your approaches to determining follow-up recommendations and your feedback about a decision support tool that is intended to make it quick and easy to determine recommendations from current practice guidelines for follow-up of polyps. Second, we would like to hear your ideas and feedback about communicating follow-up recommendations to primary care practitioners and patients.

We have a short background information form for participants. We will ask you to complete that now and then we will get started with the group.

We’d like to start by reviewing a few guidelines for this session. We are audio recording the session because we don’t want to miss anything. In summarizing the results, we will not reveal the names of individual participants.

It is important that you are aware that the discussions in the group are confidential. If you decide to participate, it is important to respect the privacy of other participants. Please be careful not to reveal the name or identifying information of anyone else in the group. We also ask you not to talk to anyone after the focus group about the opinions expressed by other participants in the focus group.

We want to hear as much as possible from everyone in the group so we will be encouraging everyone to participate. We want to encourage a wide range of opinions and we do not expect that everyone will see the issues we discuss the same way.

*A Few More Guidelines:*

- There are no right or wrong answers, only differing points of view
- We are recording, so only one person speaking at a time
- We're on a first name basis
- If possible, we ask that you turn off your phones or pagers. If you cannot and if you must respond to a call, please do so as quietly as possible and rejoin us as quickly as you can.
- My role as moderator will be to guide the discussion
- Talk to each other – not only to me as the discussion leader

Questions:

1. How do you use guidelines, recommendations or tools to determine follow up times? Probes:

Which guidelines do you use?

How often do you look up the information in the guidelines?

**Please take a look at our tool now (We will use a power point presentation, along with demonstration of use}**

2. What do you think of the tool?

Probes:

Ease of use?

reliability and validity?

Overall usefulness

3. What changes would make the tool better?

The next questions ask how you might use this tool in your practice.

4. Would you consider using this in your practice if it was accessible on a computer in your office or endoscopy suite or as an app for a smart phone?

Probes:

In what situations would you use the tool?

Would you be more likely to use the tool on a computer or smart phone?

5. How do you think this tool might alter your clinical practice?

6. Would you recommend this tool to your colleagues? Why or why not?

7 Do you think this would be beneficial for family physicians to use as well? Why or why not?

We also want to learn how we can improve the processes of ensuring that patients are aware of the timing of their follow-up appointments.

8. What can be done to improve the process of contacting patients about scheduling a follow up colonoscopy at the recommended time?

9. Would it be helpful to have a standardized or centralized procedure for informing the patient and their physician about scheduling a follow up colonoscopy, close to the time a colonoscopy is recommended? This would require many resources and therefore may not feasible soon- do you think WRHA should invest in such a system as and when resources can be allocated?

10. What else could be done to improve the follow-up of individuals with colorectal polyps and/or cancer?

11. Is there anything else you would like to add?

**Appendix IV**

**Survey of Surveillance Tool Utility in Practice**

Clinicians were asked to complete the first part of this survey (1 page) once for each of 10 patients. After the 10 patients, they were asked to complete the second part of this survey, summarizing their findings about the ease of use of the tool, whether they would use it, and suggested improvements to the tool.

Part 1

**Survey of utility of surveillance colonoscopy decision tool**

**PART ONE:** Please answer the following questions after a polyp or cancer has been discovered and after you have made your recommendation, as per your usual clinical practice.

Date of procedure: ______________________ Site of procedure: _______________________

Hospital Chart Number: ________________________

1. Did you make a recommendation immediately after the colonoscopy?
   1. Yes
      1. What was the recommendation? ______________
      2. Did your recommendation change after the pathology report was provided?
         1. Yes
         2. No
   2. No
2. What is your recommendation for interval to next colonoscopy based on your findings and the pathology report? ­­­­­­­­­­­­­­­­_______________________

**PLEASE PROCEED TO USE THE surveillance colonoscopy decision support tool AT THE WEBSITE PROVIDED:** <https://mycolonoscopy.ca/webtool/>

1. Was your recommendation the same as the recommendation from the surveillance colonoscopy decision support tool?
   1. Yes
   2. No
      1. If No, what was the reason for the discrepancy?
         1. I recall a different recommended interval
         2. I do not agree with the guidelines recommendation
         3. Other: _______________________________________
      2. Would you have changed your recommendation if you had used the tool at the time of making the recommendations?
         1. Why or why not?

___________________________________________________

Part 2

**Please print and complete this AFTER completion of all 10 surveys for PART ONE**

**Background Information Form**

This questionnaire asks a few questions about you and your professional experience and your recommendations on follow-up colonoscopy for a few scenarios. Your answers are completely confidential.

As a health care professional, are you?

O A colonoscopy performing physician – surgery

O A colonoscopy performing physician – gastroenterology

O A primary care (family) physician

O A resident in family medicine

O A resident in surgery

O A resident in gastroenterology

O Another health professional. Please specify: _________________

If you are a student how many years have you been in professional training after the bachelor’s degree? ______

If you are a health professional how many years have you been in practice after the completion of your professional training? ______

Are you: O Male O Female

Where did you do your GI/Surgery/Primary Care training? (Mark all that applies)

O US

O Canada

O Europe

O Other (please specify)

Please answer the following **ONLY** after completing all 10 surveys for part **ONE**:

1. **I felt the polyp tool to be reliable.**

( ) strongly disagree

( ) disagree

( ) neutral

( ) agree

( ) strongly agree

1. **I felt the polyp tool to be easy to use.**

( ) strongly disagree

( ) disagree

( ) neutral

( ) agree

( ) strongly agree

1. **How familiar to you or new to you is the information in the polyp tool?**

( ) very familiar

( ) familiar

( ) unsure

( ) new

( ) very new

1. **How likely would you be to use this tool on an ongoing basis in your practice?**

( ) very likely

( ) likely

( ) neutral

( ) not likely

( ) very unlikely

- 1. If you answered neutral, not likely, or very unlikely to **question 4** please select a reason:

( ) It is difficult to use

What about the tool was difficult to use? _________________________________________________________________

( ) It was not helpful

Please provide additional thoughts/feedback about why it was not helpful:

_________________________________________________________________

( ) It takes too much time to use

Is there any particular part that was time consuming?

__________________________________________________________________

( ) Other: ______________________________________________________________

______________________________________________________________________

1. **What would improve functionality of the tool for you?**

_____________________________________________________________________

1. **In what other formats would you access the tool** (e**.**g., application, hard copy):

_____________________________________________________________________

1. **If you have any further comments on the tool please add them here:**
